# Real-World Feasibility of Fibrosis-4-Based Fibrosis Risk Stratification in Patients With Type 2 Diabetes Mellitus and Metabolic Dysfunction-Associated Steatotic Liver Disease

**DOI:** 10.7759/cureus.110815

**Published:** 2026-06-14

**Authors:** Daniza A Contreras, Ammar Ibrahim

**Affiliations:** 1 Gastroenterology, Instituto Nacional de Diabetes, Endocrinología y Nutrición, Santo Domingo, DOM; 2 Faculty of Medicine, Universidad Iberoamericana (UNIBE), Santo Domingo, DOM; 3 Surgery, Instituto Nacional de Diabetes, Endocrinología y Nutrición, Santo Domingo, DOM

**Keywords:** fib-4, fibrosis-4, fibrosis risk stratification, implementation, masld, real-world study, type 2 diabetes mellitus

## Abstract

Background

Metabolic dysfunction-associated steatotic liver disease (MASLD) is highly prevalent among patients with type 2 diabetes mellitus (T2DM), a population at increased risk for advanced liver fibrosis. Current guidelines recommend the Fibrosis-4 (FIB-4) index as a first-line fibrosis risk stratification tool; however, its implementation in routine clinical practice remains uncertain.

Aim

To evaluate the real-world feasibility of FIB-4-based fibrosis risk stratification in patients with T2DM and MASLD.

Methods

We conducted a retrospective cross-sectional study including adult outpatients with T2DM and MASLD evaluated at a national referral center in Santo Domingo, Dominican Republic, between March 2022 and January 2025. Feasibility was defined as the proportion of patients with sufficient laboratory data to calculate FIB-4.

Results

A total of 397 patients were included. Only 143 patients (36.0%) had complete laboratory data required for FIB-4 calculation, while 254 (64.0%) lacked at least one required variable. AST and ALT were the most frequently missing laboratory parameters. Among patients with calculable FIB-4 values, 16 (11.2%) were classified as high risk for advanced fibrosis according to age-adjusted thresholds.

Conclusions

In this real-world cohort of patients with T2DM and MASLD, guideline-recommended FIB-4-based fibrosis risk stratification showed limited feasibility due to incomplete routine laboratory data. These findings highlight opportunities to improve the integration of standardized fibrosis risk assessment into routine diabetes care.

## Introduction

Metabolic dysfunction-associated steatotic liver disease (MASLD) is highly prevalent among patients with type 2 diabetes mellitus (T2DM), a population at increased risk for advanced liver fibrosis and liver-related complications [1-4]. Current clinical practice guidelines from the European Association for the Study of the Liver (EASL), the American Association for the Study of Liver Diseases (AASLD), and the American Diabetes Association (ADA) recommend the Fibrosis-4 (FIB-4) index as a first-line noninvasive fibrosis risk stratification tool in high-risk populations such as individuals with T2DM [5-7].

FIB-4 is calculated using routinely available laboratory parameters, including age, aspartate aminotransferase (AST), alanine aminotransferase (ALT), and platelet count, and has been validated as an effective initial screening tool for identifying patients at risk for advanced fibrosis [8,9]. Because of its simplicity and low cost, FIB-4 has been proposed as a practical strategy for large-scale implementation in routine clinical practice [10]. Although widely used, its diagnostic performance may be reduced in patients with T2DM compared with the general population [11].

Despite these recommendations, implementation of fibrosis risk stratification in routine diabetes care remains inconsistent, and real-world data regarding the feasibility of FIB-4 implementation remains limited [10-12].

The aim of this study was to evaluate the real-world feasibility of FIB-4-based fibrosis risk stratification in patients with T2DM and MASLD treated at a national referral center in the Dominican Republic.

## Materials and methods

Study design and setting

We conducted a retrospective cross-sectional study using routinely collected clinical data from adult outpatients with T2DM evaluated at the Instituto Nacional de Diabetes, Endocrinología y Nutrición (INDEN), a national referral center in Santo Domingo, Dominican Republic, between March 2022 and January 2025.

Study population

Adult patients (≥18 years) with T2DM and imaging and/or clinical findings consistent with MASLD were included.

MASLD was defined according to current international consensus criteria, requiring evidence of hepatic steatosis in the presence of at least one cardiometabolic risk factor [5,13]. In this retrospective study, diagnosis was based on imaging findings and information available in the electronic medical records.

Patients with other causes of chronic liver disease, including viral hepatitis, autoimmune liver disease, or significant alcohol consumption (>20 g/day for women and >30 g/day for men), were excluded when clinically documented.

Patients with incomplete clinical records were not excluded, since data completeness was evaluated as part of the study objective.

Data collection

Demographic and laboratory variables extracted from electronic medical records included age, sex, glycated hemoglobin (HbA1c), AST, ALT, and platelet count.

The availability of variables required for the FIB-4 index calculation was systematically assessed. Missing data were not imputed.

Fibrosis risk assessment

Fibrosis risk was assessed using the FIB-4 index, calculated according to the following formula:

$\mathrm{FIB\mathrm{-}4} = (\mathrm{Age}\,[\mathrm{years}] \times \mathrm{AST}\,[\mathrm{U/L}]) / (\mathrm{Platelet\ count}\,[10^{9}/\mathrm{L}] \times \sqrt{\mathrm{ALT}\,[\mathrm{U/L}]})$

Where AST represents aspartate aminotransferase, ALT represents alanine aminotransferase, and platelet count is expressed as ×10⁹/L. The FIB-4 score was originally developed as a noninvasive marker of liver fibrosis and has subsequently been recommended as a first-line fibrosis risk stratification tool in patients with MASLD and T2DM.

Age-adjusted thresholds were applied according to current guideline recommendations. Patients younger than 65 years were considered at increased risk of fibrosis when FIB-4 was ≥1.3, whereas a threshold of ≥2.0 was used for patients aged 65 years or older [5,6,9,14].

Statistical analysis

Statistical analyses were performed using JASP version 0.16.4 (University of Amsterdam, Amsterdam, The Netherlands). Continuous variables were expressed as mean ± standard deviation (SD), and categorical variables as frequencies and percentages. Comparisons between groups were performed using Student’s t-test or chi-square test, as appropriate. A two-sided p-value <0.05 was considered statistically significant.

## Results

Study population and data availability

A total of 397 patients with T2DM and MASLD were included in the analysis. Only 143 patients (36.0%) had complete laboratory data required to calculate the FIB-4 index, including age, AST, ALT, and platelet count. The remaining 254 patients (64.0%) lacked at least one required variable. AST and ALT were the most frequently missing laboratory parameters (Table 1).

**Table 1:** Availability of clinical and laboratory variables in the total cohort (N = 397). HbA1c: glycated hemoglobin; AST: aspartate aminotransferase; ALT: alanine aminotransferase; FIB-4: Fibrosis-4 index.

Variable	Available, n (%)	Missing, n (%)
Age	385 (97.0%)	12 (3.0%)
Sex	397 (100%)	0 (0%)
HbA1c	219 (55.2%)	178 (44.8%)
AST	171 (43.1%)	226 (56.9%)
ALT	169 (42.6%)	228 (57.4%)
Platelet count	220 (55.4%)	177 (44.6%)
Complete data for FIB-4 calculation	143 (36.0%)	254 (64.0%)

Fibrosis risk distribution among evaluable patients

Among patients with calculable FIB-4 values, 16 patients (11.2%) were classified as high risk for advanced fibrosis according to age-adjusted thresholds, while 127 patients (88.8%) were categorized as low risk.

Patients in the high-risk group were older and had higher AST levels and lower platelet counts compared with those classified as low risk (Table 2). No statistically significant differences were observed in ALT levels or HbA1c values between groups.

**Table 2:** Clinical and laboratory characteristics according to the FIB-4 risk category among evaluable patients (n = 143). Continuous variables are presented as mean ± standard deviation and categorical variables as frequency and percentage. Continuous variables were compared using Student’s t-test and categorical variables using the chi-square test. Test statistics are reported for each comparison. HbA1c: glycated hemoglobin; AST: aspartate aminotransferase; ALT: alanine aminotransferase; FIB-4: Fibrosis-4 index.

Variable	Low risk (FIB-4 <1.3) (n = 127)	High risk (FIB-4 ≥1.3) (n = 16)	Test statistic	p-value
Age, years, mean ± SD	53.89 ± 13.68	59.50 ± 12.51	t = -1.559	0.121
Female sex, n (%)	90 (70.9%)	10 (62.5%)	χ² = 0.473	0.492
AST, U/L, mean ± SD	25.07 ± 13.08	55.13 ± 36.82	t = -6.573	<0.001
ALT, U/L, mean ± SD	32.26 ± 26.83	38.73 ± 27.12	t = -0.908	0.365
HbA1c, %, mean ± SD	8.70 ± 2.41	7.81 ± 2.50	t = 1.202	0.232
Platelet count, ×10³/µL, mean ± SD	286.4 ± 63.54	236.2 ± 47.61	t = 3.048	0.003

**Figure 1:**
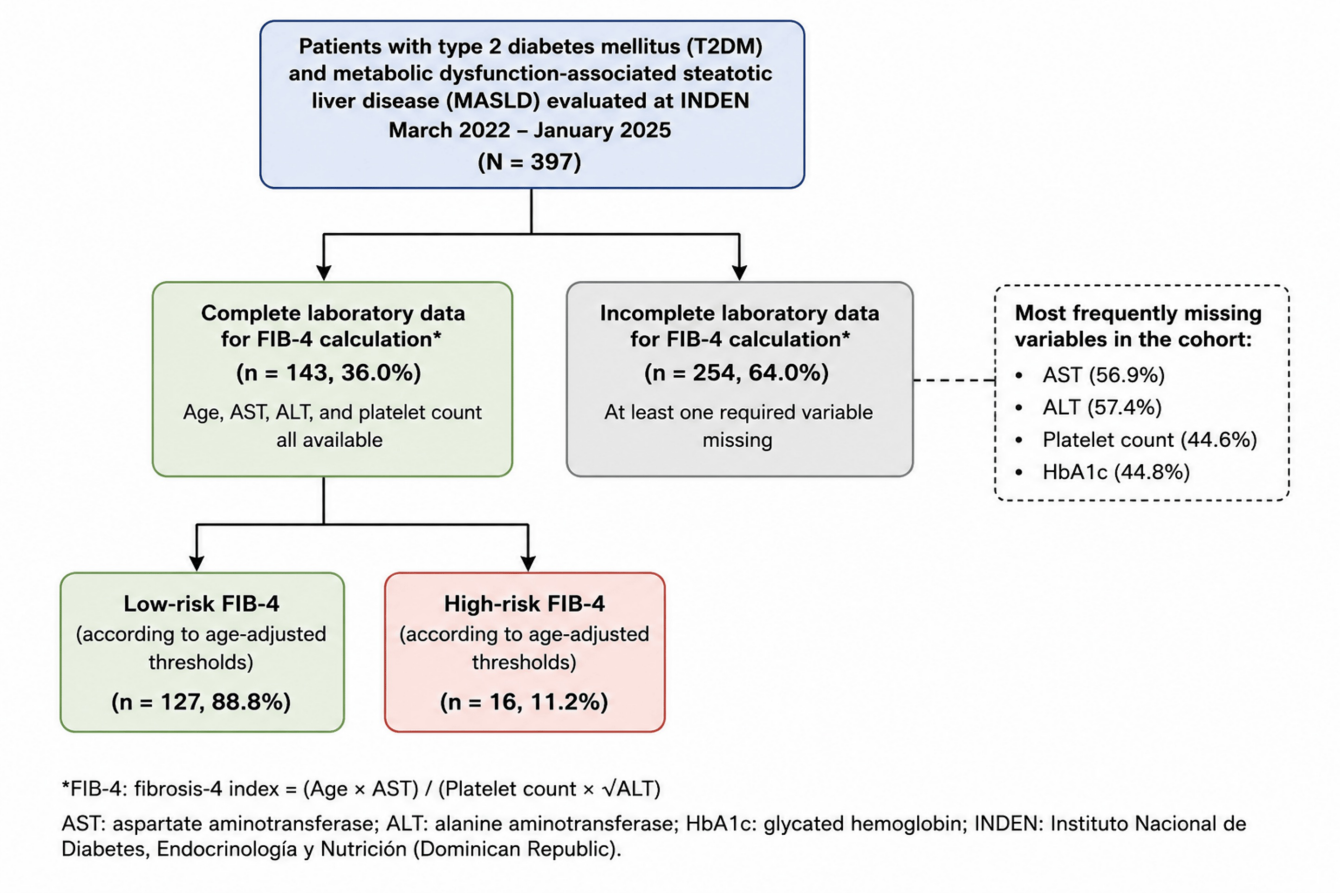
Flowchart of patient selection and feasibility of FIB-4-based fibrosis risk stratification. Patients with T2DM and MASLD evaluated at INDEN between March 2022 and January 2025 (N = 397) were included. Among them, 143 patients (36.0%) had complete laboratory data for Fibrosis-4 (FIB-4) calculation, while 254 (64.0%) lacked at least one required variable. Among evaluable patients, 127 (88.8%) were classified as low risk and 16 (11.2%) as high risk according to age-adjusted FIB-4 thresholds.

A flowchart summarizing patient selection, availability of laboratory data for FIB-4 calculation, and fibrosis risk stratification is shown in Figure 1.

## Discussion

In this real-world cohort of adult outpatients with T2DM and MASLD, only 36.0% of patients had sufficient laboratory data available to allow calculation of the FIB-4 index, despite current guideline recommendations supporting its use as a first-line fibrosis risk stratification tool in high-risk populations [5-7]. These findings suggest limited feasibility of routine FIB-4-based fibrosis risk stratification in this clinical setting. Importantly, these findings primarily reflect incomplete availability of routine laboratory parameters required for FIB-4 calculation rather than limitations of the FIB-4 tool itself.

The global burden of MASLD continues to increase, particularly among patients with obesity and T2DM, reinforcing the need for scalable fibrosis risk stratification strategies in routine clinical care [15]. Updated global consensus recommendations also support a sequential approach beginning with FIB-4 as the first-line assessment, followed by second-line noninvasive testing when indicated [16].

Current international guidelines recommend the use of simple noninvasive fibrosis assessment tools, particularly FIB-4, as the initial step in fibrosis risk stratification among patients with T2DM and MASLD [5-7,9]. Because FIB-4 relies on routinely available laboratory parameters, it has been proposed as a practical and low-cost strategy for large-scale implementation in clinical practice [8-10]. However, our findings suggest that the availability of the laboratory variables required for FIB-4 calculation remains inconsistent in routine outpatient diabetes care.

Among evaluable patients, 11.2% were classified as high risk for advanced fibrosis according to age-adjusted FIB-4 thresholds. Although fibrosis was not confirmed using elastography or liver biopsy, this finding is consistent with previous studies demonstrating a meaningful burden of fibrosis risk among patients with T2DM [4,10]. Accordingly, the distribution of FIB-4 risk categories should be interpreted as a secondary descriptive finding rather than a validated estimate of advanced fibrosis prevalence. Recent evidence has also highlighted potential limitations of FIB-4 as a standalone screening tool, particularly in high-risk populations, reinforcing the need for stepwise risk stratification strategies rather than isolated reliance on a single test [17].

This study has several limitations. First, its retrospective design resulted in substantial missing data, which limited the proportion of patients in whom FIB-4 could be calculated. However, data completeness itself represented part of the study objective and was used as a pragmatic measure of real-world feasibility. Because data were retrospectively extracted from routine electronic medical records, the specific causes underlying missing laboratory values could not be systematically determined. Nevertheless, incomplete data availability itself represented a clinically relevant finding and served as a pragmatic measure of real-world feasibility. Second, fibrosis risk was assessed using FIB-4 alone without systematic confirmation by elastography or histology. Third, the study was conducted at a single national referral center, which may limit generalizability to other settings. Finally, several potentially relevant clinical variables, including medication use and referral patterns, were not consistently available in the electronic records.

From a practical perspective, our findings suggest that improving the availability of basic laboratory parameters such as AST, ALT, and platelet count may represent an achievable strategy to facilitate implementation of guideline-recommended fibrosis risk stratification. Standardized laboratory panels and automated FIB-4 calculation integrated into electronic medical records may help improve implementation in routine outpatient care [10,11].

Recent longitudinal evidence has further strengthened the clinical value of FIB-4, demonstrating that dynamic increases in FIB-4 are associated with fibrosis progression, liver-related outcomes, cardiovascular events, and all-cause mortality among patients with MASLD. These findings support the importance of systematic FIB-4 assessment not only for initial risk stratification but also for long-term disease monitoring and prognostic evaluation [18].

This observation is consistent with the recently updated MASLD Clinical Care Pathway and recent global consensus recommendations, both of which identify implementation of fibrosis risk assessment in routine clinical practice as a key priority and support structured non-invasive fibrosis testing pathways in high-risk populations such as patients with T2DM [19,20]. Furthermore, the principal contribution of the present study lies in identifying implementation barriers and data availability challenges rather than evaluating the diagnostic performance of FIB-4 or estimating the prevalence of advanced fibrosis.

In conclusion, this study suggests limited feasibility of routine FIB-4-based fibrosis risk stratification among adult outpatients with T2DM and MASLD in this real-world setting. Improving the completeness of routinely obtained laboratory data may help facilitate the implementation of guideline-recommended fibrosis screening strategies in high-risk populations.

## Conclusions

In this real-world cohort of adult outpatients with T2DM and MASLD, only a minority of patients had sufficient laboratory data available to allow calculation of FIB-4, despite current guideline recommendations supporting its use as a first-line fibrosis risk stratification tool.

These findings suggest limited feasibility of routine FIB-4-based fibrosis risk stratification in this clinical setting and highlight opportunities to improve integration of standardized laboratory assessment into routine diabetes care pathways.

## References

[1] Targher G, Valenti L, Byrne CD (2025). Metabolic dysfunction-associated steatotic liver disease. N Engl J Med.

[2] Tilg H, Petta S, Stefan N, Targher G (2026). Metabolic dysfunction-associated steatotic liver disease in adults: a review. JAMA.

[3] Younossi ZM, Golabi P, Paik JM, Henry A, Van Dongen C, Henry L (2023). The global epidemiology of nonalcoholic fatty liver disease (NAFLD) and nonalcoholic steatohepatitis (NASH): a systematic review. Hepatology.

[4] Caussy C, Vergès B, Leleu D (2025). Screening for metabolic dysfunction-associated steatotic liver disease-related advanced fibrosis in diabetology: a prospective multicenter study. Diabetes Care.

[5] European Association for the Study of the Liver (EASL), European Association for the Study of Diabetes (EASD), European Association for the Study of Obesity (EASO) (2024). EASL-EASD-EASO clinical practice guidelines on the management of metabolic dysfunction-associated steatotic liver disease (MASLD). J Hepatol.

[6] Chalasani N, Younossi Z, Lavine JE (2018). The diagnosis and management of nonalcoholic fatty liver disease: practice guidance from the American Association for the Study of Liver Diseases. Hepatology.

[7] American Diabetes Association Professional Practice Committee (2024). Introduction and methodology: standards of care in diabetes-2024. Diabetes Care.

[8] Castera L, Friedrich-Rust M, Loomba R (2019). Noninvasive assessment of liver disease in patients with nonalcoholic fatty liver disease. Gastroenterology.

[9] European Association for the Study of the Liver (2021). EASL clinical practice guidelines on non-invasive tests for evaluation of liver disease severity and prognosis - 2021 update. J Hepatol.

[10] Davyduke T, Tandon P, Al-Karaghouli M, Abraldes JG, Ma MM (2019). Impact of implementing a “FIB-4 first” strategy on a pathway for patients with NAFLD referred from primary care. Hepatol Commun.

[11] Kanwal F, Shubrook JH, Adams LA (2021). Clinical care pathway for the risk stratification and management of patients with nonalcoholic fatty liver disease. Gastroenterology.

[12] Díaz LA, Villota-Rivas M, Barrera F, Lazarus JV, Arrese M (2024). The burden of liver disease in Latin America. Ann Hepatol.

[13] Rinella ME, Lazarus JV, Ratziu V (2023). A multisociety Delphi consensus statement on new fatty liver disease nomenclature. J Hepatol.

[14] Sterling RK, Lissen E, Clumeck N (2006). Development of a simple noninvasive index to predict significant fibrosis in patients with HIV/HCV coinfection. Hepatology.

[15] GBD 2023 MASLD Collaborators (2026). Global burden of metabolic dysfunction-associated steatotic liver disease, 1990-2023, and projections to 2050: a systematic analysis for the Global Burden of Disease Study 2023. Lancet Gastroenterol Hepatol.

[16] Younossi ZM, Kalligeros M, Wong VW (2026). Updated global consensus recommendations for risk stratification, treatment initiation, and response monitoring in metabolic dysfunction-associated steatotic liver disease. Clin Gastroenterol Hepatol.

[17] Heyens LJ, van Malde DP, Dogay Us G (2025). Is FIB-4 the right tool for screening for liver fibrosis?. Ann Hepatol.

[18] Zhou XD, Li YT, Kim SU (2026). Longitudinal changes in fibrosis markers: monitoring stiffness/fibrosis progression and prognostic outcomes in MASLD. Clin Gastroenterol Hepatol.

[19] Kanwal F, Bril F, Wong VW (2026). Clinical care pathway for the risk stratification and management of patients with metabolic dysfunction-associated steatotic liver disease. Gastroenterology.

[20] Younossi ZM, Zelber-Sagi S, Lazarus JV (2025). Global consensus recommendations for metabolic dysfunction-associated steatotic liver disease and steatohepatitis. Gastroenterology.

